# From Slow Viruses to Prions

**DOI:** 10.1371/journal.ppat.1005543

**Published:** 2016-07-07

**Authors:** Jason C. Bartz

**Affiliations:** Department of Medical Microbiology and Immunology, Creighton University, Omaha, Nebraska, United States of America; The Fox Chase Cancer Center, UNITED STATES

I entered the prion field in the early 1990s, when the nature of the infectious agent was still in dispute. At one of the very first scientific meetings I attended, a spirited argument broke out between the proponents of a viral etiology and the proponents of the protein-only (prion) etiology. One of the strong arguments made that day against the protein-only hypothesis was the observation that prion diseases displayed complex biological properties, such as strain diversity, which, at the time, were thought to be due to differences in the pathogen’s nucleic acid genome. How a protein-only agent, without a nucleic acid genome, could possess such complex biological properties was a mystery and went against biological dogma. At the time, I was a graduate student at the University of Wisconsin, and I was strongly influenced by the groundbreaking work of Howard Temin. This single conference and the knowledge that some of the basic tenants of science might need to be refined had a profound impact on my career. I have focused much of the last 25 years on understanding how prions accomplish complex biological functions in the absence of a nucleic acid genome.

Today we know that prion diseases do not have a viral etiology and are indeed caused by an infectious conformation (designated PrP^Sc^) of the normal host-encoded prion protein, PrP^C^. Prion strains are operationally defined as a phenotype of disease under a fixed set of agent and host parameters. Differences in the distribution and relative intensity of spongiform degeneration in select areas of the central nervous system are the favored criteria to distinguish strains. My early work examined interspecies transmission and adaptation of prions. What we found was that when a single prion strain was inoculated into a new host species, more than one strain would emerge. Moreover, changes in the conformation of PrP^Sc^ were predictive of which strain would emerge, lending support to the hypothesis that the conformation of PrP^Sc^ encodes strain diversity. These studies also indicated that strain interference, in which one strain interferes with the emergence of another, was an important parameter of prion adaptation to a new host species.

The ability to target PrP^Sc^ from two different strains to the same identifiable population of neurons has been essential to understanding some of the basic parameters of strain interference. This was accomplished at the start of a long-standing collaboration with a neuroscientist, Anthony (Tony) Kincaid at Creighton University. We were able to determine, in fine detail, where strain interference occurs and gain important insight into the mechanism of interference. More recent work with Tony has investigated initial prion entry and subsequent transport in the host. We have shown that different prion strains cross epithelia, are transported in blood, and spread in the nervous systems with remarkably similar temporal and spatial patterns of spread. This work suggests, in a broad sense, that strain-specific tropism of prions is quite unlike viral tropism, which is influenced by the distribution of viral receptors. Instead, prions appear to be transported to a variety of cells, and the balance between replication, clearance, and toxicity may govern prion tropism.

Another unexpected property of prions is that they can persist and remain infectious even after years in the environment. A chance phone call from an environmental engineer from the University of Nebraska, Shannon Bartelt-Hunt, has led to a fruitful collaboration and a series of important observations regarding this issue. Working with Shannon, we have identified that the soil type, the matrix that contains prions, and the strain of prion influences the binding of prions to soil. Additionally, this work demonstrated that weathering can partially inactivate prions and that PrP^Sc^ bound to surfaces has a strong influence on this process. I have been fortunate to collaborate with many talented investigators, and I have found that interdisciplinary collaborations have moved this work forward in interesting and sometimes unexpected directions.

Prion research has provided the groundwork for important observations in other areas of biology. In yeast and fungi, there are numerous reports of two conformations of the same protein having different cellular functions. Importantly, the self-propagating (prion) conformation of the protein can alter the normal conformation of the protein into a misfolded prion conformation, as is seen in prion diseases of animals. Unlike prion diseases in animals, which are uniformly fatal, yeast prions can serve a useful function to the cell and can be thought of as a protein-based epigenetic mode of inheritance. Recent work suggests that other protein misfolding diseases share much in common with prion diseases. For example, the formation of amyloid in Alzheimer’s disease and Lewy bodies in Parkinson’s disease are thought to occur by a prion-like mechanism. Moreover, the spread of neuropathology in Parkinson’s disease and amyotrophic lateral sclerosis follows neuroanatomical pathways that are strikingly similar to those that we have reported in our studies of prion diseases. Also, the formation of α-synuclein fibrils with distinct biochemical properties that result in distinct patterns of pathology in Parkinson’s disease is reminiscent of what we and others have reported with prion strains. These are some of the unexpected and potentially significant findings of prion disease research that have implications for human health that extend far beyond the traditional boundaries of this relatively rare class of neurodegenerative diseases.

**Image 1:**
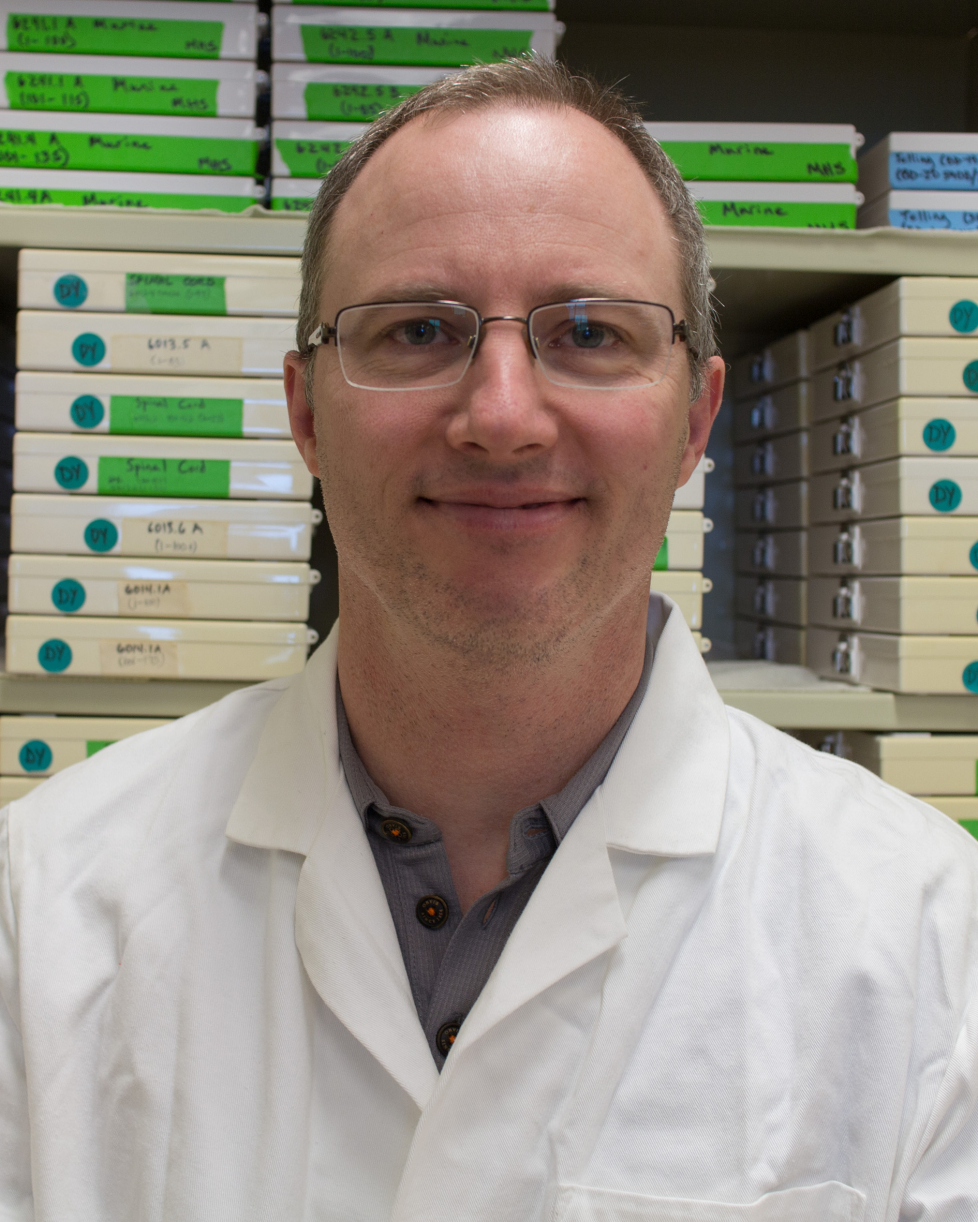
Jason C. Bartz.

